# Gene panel testing for breast cancer should not be used to confirm syndromic gene associations

**DOI:** 10.1038/s41525-018-0071-6

**Published:** 2018-11-26

**Authors:** D. Gareth Evans, Sacha J. Howell, Ian M. Frayling, Juha Peltonen

**Affiliations:** 1 Manchester Centre for Genomic Medicine, Central Manchester University Hospitals NHS Foundation Trust, Manchester, M13 9WL UK; 2 Division of Evolution and Genomic Sciences, School of Biological Sciences, Faculty of Biology, Medicine and Health, University of Manchester, Manchester Academic Health Science Centre, Manchester, UK; 3 The Christie NHS Foundation Trust, Wilmslow Road, Manchester, M20 4BX UK; 4 Division of Cancer Sciences, Faculty of Biology, Medicine and Health, University of Manchester, Manchester Academic Health Science Centre, Manchester, UK; 5 All-Wales Medical Genetics Service, Cardiff & Vale University Health Board, University Hospital of Wales, Cardiff, CF14 4XW UK; 6 Institute of Medical Genetics, Division of Cancer & Genetics, School of Medicine, Cardiff University, Cardiff, CF14 4XN UK; 7 Department of Cell Biology and Anatomy, Institute of Biomedicine, University of Turku, Turku, Finland

In recent years, there has been an explosion in the use of multi-gene panels to test for cancer predisposition often utilising large panels across many tumour types. More recently, the results of these tests have been used as a form of case control study to assess genes for cancer associations, especially with breast cancer. Four recent articles based on multi-gene panel testing, published in high impact oncology journals, have concluded that there are no associations between pathogenic variants in three syndromic genes (*NF1, PTEN* and *STK11*) and breast cancer risk.^1–4^ Whilst these analyses have identified potential new gene associations, the negative results concerning syndromic associations should be tempered. In the first of their three articles, Ambry’s 21 panel gene test^1^ was evaluated in 41,611 consecutively tested white women with breast cancer. In the second,^2^ 9639 patients with breast cancer were assessed, whilst the third assessed the risk of triple negative breast cancer in 8753 women.^3^ Whilst two studies used control data from the ExAc database^1,3^ the second used a combination of controls tested for non-cancer indications.^2^ The larger initial study^1^ identified *NF1* gene variants in 0.1% compared to 0.11% in ExAc controls. No control frequency was provided for the second study^2^ although a frequency in cases of around 0.15% was said to be non-significant.^2^ The third study also found a frequency of 0.15% in triple negative breast cancer which was also non-significant.^3^ The first two studies effectively excluded *BRCA1* and *BRCA2* as they confirmed that there had been a high degree of pre-testing for these genes. The first also excluded what they ‘termed’ ‘syndromic’ genes including *PTEN, CDH1* and *TP53*. However, it is unclear why neurofibromatosis 1, caused by pathogenic variants in the *NF1* gene, was not also excluded as being syndromic, as it is far more recognisable from patient characteristics than even PTEN hamartoma syndrome.^5^

There are two main flaws in the conclusions that *NF1* is not associated with breast cancer. The first is that panel testing for breast cancer is selective based on family history, age and patient/clinician choice. A clinician who already has an ‘explanation’ for a breast cancer in a patient with NF1 is unlikely to send off for a gene panel. Indeed the syndromic learning problems associated with NF1 may also preclude gainful employment and thus reimbursement for panel testing. The link to breast cancer based on cohort studies is now irrefutable with six studies reporting Odds Ratios of 4–11 fold for NF1 women aged <50 years of age.^6,7^ The three largest series published were from population-based series using population cancer registries and genetic registers^6,7^ that are much less likely to have ascertainment bias than hospital-based series. The vast majority of NF1 cases reported with breast cancer were known to have NF1 prior to breast cancer diagnosis.^6,7^ Furthermore, driver *NF1* pathogenic variants have been identified in the Cancer Genome Atlas and from NF1 patients are associated with higher tumour grade and Human Epidermal Growth Factor Receptor 2 (HER2) overexpression,^8,9^ further evidence against a chance association. Indeed the breast cancers occurring in NF1 cases reported in the cohort studies are also more aggressive with very poor survival compared to controls.^6,7^ If there was an ascertainment bias for breast cancer prior to NF1 diagnosis this would lead to an immortal survivor bias that would artificially improve survival.^6^

The link with breast cancer and NF1 has been established since at least 2007.^7^ All of the reported panel tests were performed since March 2012,^1–4^ after four of the cohort studies had reported showing a probable causal association with breast cancer and NF1.^7^ The authors of the Ambry reports clearly admit that there was already preselection for BRCA testing.^1–3^ If a clinician wished to test for *NF1* they would use a substantially more sensitive targeted strategy incorporating an RNA-based approach than a panel.^8^ Many pathogenic variants would be missed by an exonic DNA-based strategy as deep intronic splicing variants are common as well as missense variants that cause splicing that would otherwise be reported as variants of unknown significance.^10^ A similar concern should also be expressed for the apparent lack of association with *PTEN* in the second and third Ambry studies^2,3^ as these were not powered to assess the lower frequency of *PTEN* pathogenic variants and suffer from the same issue of lack of requirement for a panel test when an explanation for breast cancer was already present. The same criticism can be put forward for the absence of *STK11* variants being identified in 2000 familial breast cancer samples tested in the LIFEPOOL study.^4^ Peutz-Jeghers disease caused by *STK11* variants is easily identified by peri-oral pigmentation and usually presents symptomatically in early life with multiple intestinal polyps^11^ (Table 1).

**Table 1:** Syndromic genes identifiable from clinical features in a single woman that increase breast cancer risk from cohort studies

Gene	Syndrome	Birth incidence	Clinical features	Breast cancer risk to 50 years/lifetime	Other malignancy risk
*NF1*	Neurofibromatosis 1	1 in 2000–2500	*Café au lait* Cutaneous neurofibromas Iris Lisch nodules	10%/20%	Malignant peripheral nerve sheath tumour, glioma
*PTEN*	PTEN hamartoma syndrome (Cowden)^5^	1 in 100,000–200,000	Macrocephaly, mucocutaneous lesions (e.g. Trichilemmomas)	50%/85%	Thyroid, endometrial
*STK11*	Peutz-Jeghers^11^	1:25,000 to 1:280,000	Peri-oral pigmentation, hamartomatous bowel polyps	Nk/37–55%	Colorectal, stomach, small bowel, ovary, cervix, pancreas, testes

The second potential flaw is the use of ExAc controls for *NF1*. The very high frequency of apparent pathogenic variants of 1/900 is simply not consistent with the estimates of birth incidence from highly ascertained populations of between 1 in 2000–2600.^12,13^ Due to early deaths in NF1 patients, prevalence in an adult population is nearer 1 in 3000–4500.^12,13^ It is therefore unclear whether this high estimate in controls is due to over assessment of pathogenic variants, selection for children to have exomes with NF1 features or due to some variants being silent clinically. The latter is unlikely as among cases investigated further by Ambry although only half clearly had NF1 from a review of the proforma and oncology notes, but expert review confirmed clinical features consistent with NF1 in the cases a with full constitutional *NF1* variant without a known diagnosis that were seen by an expert.^14^ This highlights an additional issue with publishing results of commercial panel testing in that these can be heavily reliant on accurate information from proformas which are likely to be only partially completed in many instances. Additionally of those without a known diagnosis, 16/42 (38%) were mosaic for the *NF1* variant and may not have had clinical features. No distinction is made in the Ambry reports about comparing mosaic and non-mosaic frequencies of *NF1* variants.^1–3^ Indeed it might be expected that mosaic involvement of NF1 would be less likely to result in breast cancer and this was reflected by an older age at testing of 67 years compared to 49 years for constitutional cases.^14^

Case control analysis is arguably the most informative method to identify gene–cancer associations as it also provides confirmation of the level of any increased risk. Ideally the ‘cases’ should be derived from a truly unselected series of individuals with relevant cancer. Similarly the controls should come from the same population, and can be either true ‘population’ controls from representative, unselected and ideally age-matched individuals, or ‘super’ controls (older/age-matched individuals known not to have the malignancy being investigated). Particularly for rare diseases (present in less than 1 in 2000 individuals), very large case control series are required to confirm moderate risk elevations of only 2–3 fold. Whilst traditional methods such as positional cloning from family linkage were used for identifying the *BRCA1* and *BRCA2* genes in 1994 and 1995, most other breast cancer predisposition genes were identified as causal from candidate gene approaches using case control series often enhanced by using familial samples. However, even these have identified potentially spurious associations, because analyses of breast/ovarian cancer families have identified a real association with ‘ovarian’ cancer, but a potentially false association with breast cancer.^15,16^ In particular, initial breast cancer associations with *RAD51C, RAD51D* and *BRIP1* were later called into question with breast cancer-specific analysis.^15,16^ Hence, these three genes are not on the UK’s National Health Service breast cancer panel,^17^ but they do appear on most commercial ‘breast cancer’ specific panels. Other DNA repair gene associations, such as for *ATM, CHEK2* and *PALB2*, have been well validated in multiple cohort studies and added to breast cancer-specific gene panels.^17^ In contrast to the aforementioned genes which have no recognisable syndromic phenotype for an individual, the phenotypes from germline pathogenic variants in *NF1, PTEN* and *STK11* (Table 1) are usually easily recognisable in single individuals and there are well-validated diagnostic criteria which allow a clinical diagnosis without the need for molecular confirmation.^5,11^

In conclusion, the use of commercial multi-gene panels to confirm syndromic associations with cancer is problematic and the results of these should not in particular be used to refute the far better evidence from cohort studies^6,7^ backed up by biology^6,8,9^ in NF1. Whilst panel tests appear to be a useful agnostic test for cancer associations with non-syndromic genes, they are not when considering easily recognisable syndromes, as these create biases in selection against any association and may also contaminate controls. The only way to robustly assess for links with syndromes in a case control study is for ALL patients with breast cancer to be tested on a population basis with appropriate controls tested of a similar age, also without selection.
